# Teaching Cardiac Auscultation: Effectiveness of Virtual Simulation-Based Training on Improving Cardiac Auscultatory Skills in Post-graduate Trainees in the COVID-19 Era

**DOI:** 10.7759/cureus.80873

**Published:** 2025-03-20

**Authors:** Melissa Hidalgo, Sophia Navajas, Michael A Chizner

**Affiliations:** 1 Cardiovascular Medicine, Broward Health Medical Center, Fort Lauderdale, USA; 2 Internal Medicine, Broward Health North, Deerfield Beach, USA

**Keywords:** cardiac auscultation, harvey, heart sounds, murmurs, virtual simulation

## Abstract

Background: The art of cardiac auscultation is an essential clinical skill being lost amid growing reliance on modern technology and the steady decline in bedside teaching in the wake of the COVID-19 pandemic.

Objectives: This study aimed to assess the cardiac auscultatory skills of post-graduate trainees within a single-center design before and after a virtual simulation-based training course and to determine the effectiveness of this teaching method on improving diagnostic proficiency.

Methods: Fifty-eight residents attended a seven-hour virtual simulation-based training course on cardiac auscultation, one hour per month, during their daily graduate medical education sessions. Residents completed a pre-test and post-test on their ability to diagnose 12 important heart sounds and murmurs produced via Harvey, the Cardiology Patient Simulator. Scores were compared using a two-tailed test. Residents also completed a feedback survey before and after the course.

Results: All 58 residents completed the course as well as the pre- and post-quiz. Of the 12 auscultatory events analyzed, an overall pre-test identification score of 27.44% and a post-test score of 39.08% (an increase of 42%) were observed, with a response rate of 100%. This represents a statistically significant improvement in diagnostic proficiency (p<0.001).

Conclusions: Although not a substitute for in-person bedside teaching, a virtual simulation-based training course is an effective method of teaching cardiac auscultatory skills and improving the diagnostic proficiency of post-graduate trainees in the COVID-19 era.

## Introduction

Despite advances in modern technology, cardiac auscultation remains a valuable and cost-effective diagnostic tool in clinical evaluation, yet it faces decline amid modern reliance on technology and reduced bedside teaching, especially exacerbated by the COVID-19 pandemic [1,2].

Many training programs lack structured teaching for cardiac auscultation, resulting in diminishing skills among medical trainees [3-12]. The pandemic's contact restrictions further hindered in-person "hands-on" patient interactions, limiting the improvement of clinical examination skills [13].

Virtual simulation offers a potential solution [14], yet its effectiveness for post-graduate trainees during COVID-19 remains underexplored. Even in this age of modern technology, cardiac auscultation has the advantage of being a readily available and cost-effective diagnostic technique that allows the prompt recognition of many important cardiac diseases [1,8,15-20]. However, during the COVID-19 pandemic, in-person restrictions lead to an urgent need for safe and effective alternative training methods for learning and teaching bedside clinical skills [21,22]. 

Accordingly, we set out to explore whether utilizing a virtual simulation-based training course would enhance cardiac auscultatory skills in post-graduate trainees within a single-center design and to determine the effectiveness of this innovative clinical teaching method in improving diagnostic proficiency during the COVID-19 era.

## Materials and methods

Setting and participants

Forty-three internal medicine residents (PGY-1, PGY-2, PGY-3) and 15 transitional year residents (PGY-1) from Broward North Medical Center participated from November 2023 to May 2024.

Interventions

Fifty-eight residents attended a seven-hour virtual simulation-based training course on cardiac auscultation using video demonstrations of Harvey, the Cardiology Patient Simulator. The course was held one hour per month over seven months during their daily graduate medical education sessions. It was taught by an experienced clinician-educator (MAC) via Microsoft Teams (Microsoft Corporation, Redmond, Washington, United States). The teaching sessions were based on video demonstrations using Harvey, along with the experience of the instructor as to how one arrives at a correct diagnosis for each heart sound and murmur based on their characteristic findings (timing, location, intensity, duration, frequency, shape, quality, and variation with respiration). The residents completed pre-test and post-test examinations on their ability to diagnose a wide variety of cardiac conditions. All participants were initially tested on 12 clinically important heart sounds and murmurs, produced via Harvey, the Cardiology Patient Simulator, including splitting of the second heart sound, S3 and S4 gallops, mid-systolic click, along with the murmurs of aortic stenosis, aortic regurgitation, mitral stenosis, mitral regurgitation, tricuspid regurgitation, patent ductus arteriosus, innocent systolic murmur, and the pericardial friction rub. All auscultatory events were presented in a randomized fashion. Each participant was told where the stethoscope had been placed on the chest wall. Test takers had the opportunity to listen for 20 seconds and were asked to arrive at a diagnosis based strictly on auscultation. Of note, no other historical or pertinent clinical information was provided. Sums of correct and incorrect answers for all residents were determined for each event on both the pre-test and post-test examinations. A feedback survey was also taken by all residents before and at the end of the virtual simulation-based training course.

Outcomes measured

Pre-test and post-test evaluations assessed residents' ability to diagnose 12 important heart sounds and murmurs using Harvey including splitting of the second heart sound, S3 and S4 gallops, mid-systolic click, aortic stenosis, aortic regurgitation, mitral stenosis, mitral regurgitation, tricuspid regurgitation, innocent murmur, continuous murmur, and the pericardial friction rub. Feedback surveys were also conducted.

Analysis of outcomes

Data was entered into IBM SPSS Statistics for Windows, Version 30.0.0.0 (Released 2024; IBM Corp., Armonk, New York, United States) for all pre-test and post-test examinations, using a binary conversion of 1 for pre-test and 0 for post-test. The null hypothesis stated that the mean test scores of the pre-test and post-test would not differ. Results were analyzed using a two-tailed test with a significance level of p<0.001.

IRB statement

This project was exempt from review by the Institutional Review Board (IRB) of Broward Health.

## Results

All 58 residents attended the seven-hour lecture and completed both the pre- and post-quiz. The study observed a statistically significant improvement in diagnostic proficiency. Pre-test scores averaged 27.44% (3.29 out of 12 correctly identified heart sounds), while post-test scores averaged 39.08% (4.69 out of 12), reflecting a 42% increase in accuracy. An independent samples t-test was conducted to compare pre-test and post-test scores. Levene's test indicated that the assumption of equal variances was met (F(1,114)=0.771; p=0.382). The results showed a statistically significant difference between the pre-test (M=3.29; SD=1.533) and post-test (M=4.69; SD=1.729) scores (t(114)=-4.60; p<0.001). The mean difference was -1.397 (99% CI (-2.191, -0.602)), indicating a significant improvement in scores after the intervention as shown in Table 1.

**Table 1:** Levene's test for equality of variance

Independent samples test									
Test	F	Sig.	t	df	One-sided p	Two-sided p	Mean difference	Std. error difference	99% confidence interval (lower, upper)
Equal variances assumed	0.771	0.382	-4.602	114	<0.001	<0.001	-1.397	0.303	(-2.191, -0.602)
Equal variances not assumed			-4.602	112.393	<0.001	<0.001	-1.397	0.303	(-2.192, -0.601)
Group statistics									
Group	N	Mean	Std. deviation	Std. error mean					
Pre-test	58	3.29	1.533	0.201					
Post-test	58	4.69	1.729	0.227					

Detailed results

**Figure 1:**
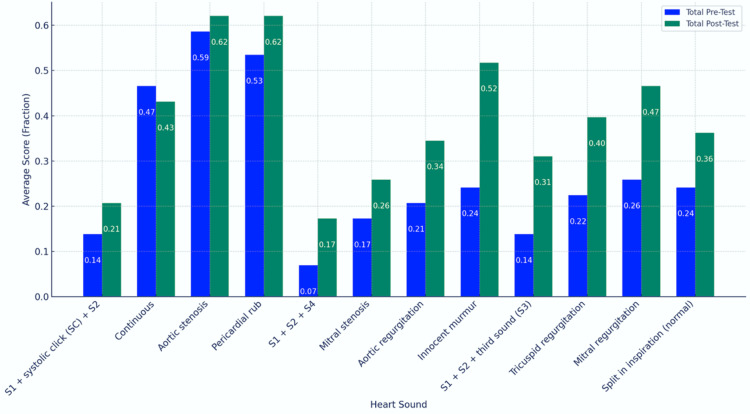
Total pre-test and post-test averages for the 12 tested heart sounds with blue indicating the pre-test average scores and green indicating the post-test average scores

Figure 1 depicts the total pre-test and post-test averages for the 12 tested heart sounds. The following are the pre-test scores: continuous murmur (47%), S1+systolic click+S2 (14%), aortic stenosis (59%), pericardial rub (53%), mitral stenosis (17%), S1+S2+S4 (7%), innocent murmur (24%), aortic regurgitation (21%), S1+S2+third sound (14%), tricuspid regurgitation (22%), physiological splitting of S2 (24%), and mitral regurgitation (26%). In contrast, the following are the post-test scores: continuous murmur (43%), S1+systolic click+S2 (21%), aortic stenosis (62%), pericardial rub (62%), mitral stenosis (26%), S1+S2+S4 (17%), innocent murmur (52%), aortic regurgitation (34%), S1+S2+third sound (31%), tricuspid regurgitation (22%), physiological splitting of S2 (36%), and mitral regurgitation (47%).

**Figure 2:**
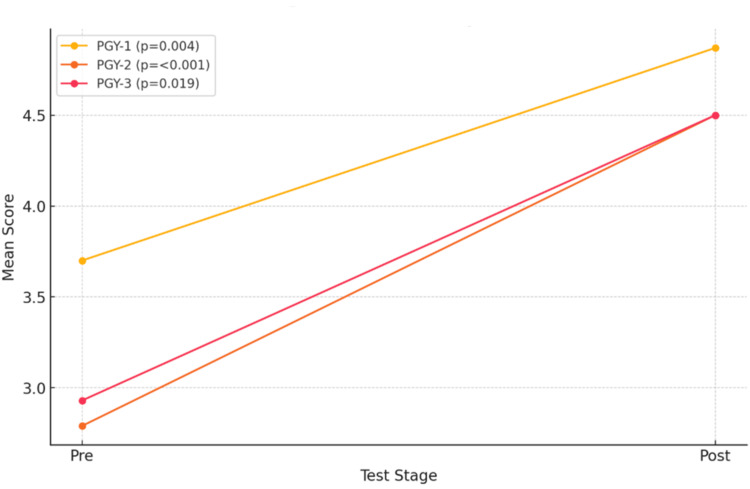
Pre-test to post-test comparison per PGY level, with yellow indicating pre-test to post-test average scores for PGY-1 level, orange indicating pre-test to post-test average scores for PGY-2 level, and pink indicating pre-test to post-test average scores for PGY-3 level

The average pre-quiz score for PGY-1 residents was 33%, which improved to 41.6% on the post-quiz assessment. For PGY-2 and PGY-3 residents, the average pre-quiz score was 25%, with a similar improvement to 41.6% post-quiz as shown in Figure 2.

Feedback surveys indicated high levels of acceptance and satisfaction, with increased confidence in recognizing heart sounds and murmurs.

## Discussion

Our findings indicate significant improvement in cardiac auscultatory skills following the virtual simulation-based training course. This study supports existing literature that cardiac auscultatory proficiency among today's post-graduate trainees is indeed poor and highlights the value of simulation-based education in improving clinical skills [5,14].

In comparison with existing literature, our participants were able to correctly identify a variety of auscultatory events, such as the murmur of mitral regurgitation (47%), aortic stenosis (62%), and pericardial friction rub (62%) with a higher degree of accuracy as shown in Table 2. However, certain auscultatory events, such as mitral stenosis (26%), aortic regurgitation (34%), and the S4 gallop (17%), continued to present a diagnostic challenge, as was also observed in prior studies [10,19,23,24].

**Table 2:** Pre- and post-test % score by heart sound

Heart sound	Pre-test PGY-1 (%)	Post-test PGY-1 (%)	Pre-test PGY-2 (%)	Post-test PGY-2 (%)	Pre-test PGY-3 (%)	Post-test PGY-3 (%)	Total pre-test (%)	Total post-test (%)
S1+systolic click (SC)+S2	13.3	20	14.3	14.3	14.3	28.6	13.8	20.7
Continuous murmur	53.3	40	42.9	57.1	35.7	35.7	46.6	43.1
Aortic stenosis	66.7	73.3	57.1	50	42.9	50	58.6	62.1
Pericardial rub	50	56.7	64.3	100	50	42.9	53.4	62.1
S1+S2+S4	3.3	10	0	35.7	21.4	14.3	6.9	17.2
Mitral stenosis	23.3	30	7.1	7.1	14.3	35.7	17.2	25.9
Aortic regurgitation	23.3	36.7	21.4	28.6	14.3	35.7	20.7	34.5
Innocent murmur	26.7	50	21.4	42.9	21.4	64.3	24.1	51.7
S1+S2+third sound (S3)	13.3	33.3	0	28.6	28.6	28.6	13.8	31
Tricuspid regurgitation	30	53.3	7.1	21.4	21.4	28.6	22.4	39.7
Mitral regurgitation	40	50	7.1	50	14.3	35.7	25.9	46.6
Split in inspiration (normal)	26.7	33.3	28.6	28.6	14.3	50	24.1	36.2

Cardiac auscultation is a difficult task that often necessitates the detection of auscultatory events at the threshold of audibility. S3 and S4 gallops, for example, are low in frequency and faint in intensity. Inexperienced examiners cannot reliably agree on their presence [24]. This may explain the interobserver variability in the detection of the S3 or S4 gallop sounds noted in previous studies and the poor recognition at both the beginning and end of our study (although there was a statistically significant improvement). Furthermore, the inability to position the patient in the left lateral decubitus position may contribute to the poor ability of our test takers to detect gallop sounds based on auscultation alone.

Perhaps the poor recognition of certain auscultatory findings such as the mid-systolic click of mitral valve prolapse (~21%) and the diastolic murmurs of mitral stenosis (26%) and aortic regurgitation (~34%) after course completion was due to the inability of test takers to palpate the arterial pulse and/or apical impulse or "inch" the stethoscope. Consequently, they were unable to differentiate systole from diastole by auscultation alone. 

A unique feature of our study was the use of a virtual simulation-based training platform using the transmission of heart sounds and murmurs from the Cardiology Patient Simulator, a proven system to teach and learn bedside clinical skills that transfer to real patients [25]. Although simulation-based training has been shown to be of value in learning cardiac auscultation [25-28], the usefulness of a virtual platform in teaching these auscultatory skills to post-graduate trainees in the era of COVID-19 has not been previously appreciated. Worthy of mention, the delivery of simulation training was also seriously affected during the pandemic; therefore, an innovative virtual platform for teaching and learning essential clinical skills was needed and implemented.

Relevant datasets such as the pre- and post-scores per PGY levels, the entire sample pre- and post-test score T-statistics, and the pre- and post-test score T-statistics in residents per PGY levels are shown in the Appendices.

Limitations of our study include the single-center design of post-graduate trainees, which may decrease the generalizability to other contexts and healthcare professions. Furthermore, our results may not fully capture the variability in resources, patient demographics, or clinical protocols that exist in other institutions or geographic regions. In addition, the size of our study may amplify the reported results. The training intervention was short duration (seven hours over a seven-month period), and the lack of long-term retention assessment limits the generalizability of the findings. The inability of test takers in our study to do a physical exam may have also represented a limitation of our testing method.

Further research is needed to evaluate the long-term retention of skills and the effectiveness of integrating virtual simulation with traditional bedside teaching.

## Conclusions

Our study demonstrated that while virtual simulation-based training cannot replace traditional, in-person bedside teaching, it significantly improves cardiac auscultatory skills in post-graduate trainees. This innovative, safe, and effective method of virtual teaching can address the challenges of bedside teaching in the post-COVID-19 era. Therefore, it should be integrated into the curriculum and considered a dedicated component of residency training programs.

## Appendices

**Table 3:** Pre- and post-scores: PGY-1 level

Pre-score	Post-score	Initials
4/12	8/12	PGY-1
2/12	7/12	PGY-1
1/12	4/12	PGY-1
4/12	7/12	PGY-1
4/12	3/12	PGY-1
4/12	5/12	PGY-1
5/12	4/12	PGY-1
4/12	6/12	PGY-1
4/12	3/12	PGY-1
3/12	7/12	PGY-1
0/12	2/12	PGY-1
4/12	3/12	PGY-1
4/12	6/12	PGY-1
3/12	6/12	PGY-1
6/12	4/12	PGY-1
6/12	3/12	PGY-1
4/12	3/12	PGY-1
3/12	3/12	PGY-1
6/12	5/12	PGY-1
4/12	1/12	PGY-1
3/12	5/12	PGY-1
1/12	5/12	PGY-1
5/12	7/12	PGY-1
5/12	7/12	PGY-1
3/12	4/12	PGY-1
2/12	5/12	PGY-1
4/12	5/12	PGY-1
2/12	6/12	PGY-1
6/12	6/12	PGY-1
5/12	6/12	PGY-1

**Table 4:** Pre- and post-scores: PGY-2 level

Pre-score	Post-score	Initials
3/12	6/12	PGY-2
4/12	5/12	PGY-2
3/12	4/12	PGY-2
5/12	4/12	PGY-2
3/12	6/12	PGY-2
2/12	3/12	PGY-2
2/12	4/12	PGY-2
1/12	6/12	PGY-2
1/12	4/12	PGY-2
4/12	4/12	PGY-2
4/12	4/12	PGY-2
0/12	3/12	PGY-2
3/12	6/12	PGY-2
4/12	4/12	PGY-2

**Table 5:** Pre- and post-scores: PGY-3 level

Pre-score	Post-score	Initials
5/12	4/12	PGY-3
5/12	2/12	PGY-3
5/12	2/12	PGY-3
1/12	3/12	PGY-3
4/12	5/12	PGY-3
3/12	5/12	PGY-3
2/12	10/12	PGY-3
3/12	3/12	PGY-3
3/12	3/12	PGY-3
3/12	5/12	PGY-3
2/12	8/12	PGY-3
2/12	5/12	PGY-3
0/12	3/12	PGY-3
3/12	5/12	PGY-3

**Table 6:** Entire sample pre- and post-test score T-statistics

Independent samples test									
Test	F	Sig.	t	df	One-sided p	Two-sided p	Mean difference	Std. error difference	99% confidence interval (lower, upper)
Equal variances assumed	0.771	0.382	-4.602	114	<0.001	<0.001	-1.397	0.303	(-2.191, -0.602)
Equal variances not assumed			-4.602	112.393	<0.001	<0.001	-1.397	0.303	(-2.192, -0.601)
Group statistics									
Group	N	Mean	Std. deviation	Std. error mean					
Pre-test	58	3.29	1.533	0.201					
Post-test	58	4.69	1.729	0.227					

**Table 7:** Pre- and post-test score T-statistics in PGY-1 residents

Independent samples test									
Variance assumption	F	Sig.	t	df	One-sided p	Two-sided p	Mean difference	Std. error difference	99% confidence interval (lower, upper)
Equal variances assumed	1	0.321	-2.757	58	0.004	0.008	-1.167	0.423	(-2.294, -0.040)
Equal variances not assumed			-2.757	57.135	0.004	0.008	-1.167	0.423	(-2.294, -0.039)
Group statistics									
Group	N	Mean	Std. deviation	Std. error mean					
Pre-test	30	3.7	1.535	0.28					
Post-test	30	4.87	1.737	0.317					

**Table 8:** Pre- and post-test score T-statistics in PGY-2 residents

Independent samples test									
Variance assumption	F	Sig.	t	df	One-sided p	Two-sided p	Mean difference	Std. error difference	99% confidence interval (lower, upper)
Equal variances assumed	0.641	0.431	-3.575	26	<0.001	0.001	-1.714	0.48	(-3.047, -0.382)
Equal variances not assumed			-3.575	24.361	<0.001	0.002	-1.714	0.48	(-3.054, -0.375)
Group statistics									
Group	N	Mean	Std. deviation	Std. error mean					
Pre-test	14	2.79	1.424	0.381					
Post-test	14	4.5	1.092	0.292					

**Table 9:** Pre- and post-test score T-statistics in PGY-3 residents

Independent samples test									
Variance assumption	F	Sig.	t	df	One-sided p	Two-sided p	Mean difference	Std. error difference	99% confidence interval (lower, upper)
Equal variances assumed	1.383	0.25	-2.182	26	0.019	0.038	-1.571	0.72	(-3.573, 0.430)
Equal variances not assumed			-2.182	22.609	0.02	0.04	-1.571	0.72	(-3.597, 0.454)
Group statistics									
Group	N	Mean	Std. deviation	Std. error mean					
Pre-test	14	2.93	1.492	0.399					
Post-test	14	4.5	2.245	0.6					
